# Aspalathin-rich green rooibos tea in combination with glyburide and atorvastatin enhances lipid metabolism in a *db/db* mouse model

**DOI:** 10.3389/fcdhc.2022.963489

**Published:** 2022-10-03

**Authors:** Oelfah Patel, Christo J. F. Muller, Elizabeth Joubert, Bernd Rosenkranz, Johan Louw, Charles Awortwe

**Affiliations:** ^1^ Biomedical Research and Innovation Platform (BRIP), South African Medical Research Council (MRC), Tygerberg, South Africa; ^2^ Division of Clinical Pharmacology, Department of Medicine, Faculty of Medicine and Health Sciences, University of Stellenbosch, Tygerberg, South Africa; ^3^ Centre for Cardio-metabolic Research in Africa, Division of Medical Physiology, Faculty of Medicine and Health Sciences, Stellenbosch University, Tygerberg, South Africa; ^4^ Department of Biochemistry and Microbiology, University of Zululand, KwaDlangezwa, South Africa; ^5^ Department of Food Science, Stellenbosch University, Matieland, South Africa; ^6^ Post-Harvest and Agro-Processing Technologies, Agricultural Research Council, Infruitec-Nietvoorbij, Stellenbosch, South Africa

**Keywords:** glyburide, rooibos, atorvastatin, lipid metabolism, fasting plasma glucose, type 2 diabetes,

## Abstract

Rooibos (*Aspalathus linearis*), an indigenous South African plant and its major flavonoid, aspalathin, exhibited positive effects on glycemia and dyslipidemia in animal studies. Limited evidence exists on the effects of rooibos extract taken in combination with oral hypoglycemic and lipid-lowering medications. This study investigated the combined effects of a pharmaceutical grade aspalathin-rich green rooibos extract (GRT) with the sulfonylurea, glyburide, and atorvastatin in a type 2 diabetic (*db/db*) mouse model. Six-week-old male *db/db* mice and their nondiabetic lean *db^+^* littermates were divided into 8 experimental groups (n=6/group). *Db/db* mice were treated orally with glyburide (5 mg/kg bodyweight), atorvastatin (80 mg/kg bodyweight) and GRT (100 mg/kg bodyweight) as mono- and combination therapies respectively, for 5 weeks. An intraperitoneal glucose tolerance test was conducted at 3 weeks of treatment. Serum was collected for lipid analyses and liver tissues for histological examination and gene expression. A significant increase in the fasting plasma glucose (FPG) of the *db/db* mice compared to their lean counterparts (from 7.98 ± 0.83 to 26.44 ± 1.84, p < 0.0001) was observed. Atorvastatin reduced cholesterol (from 4.00 ± 0.12 to 2.93 ± 0.13, p < 0.05) and triglyceride levels (from 2.77 ± 0.50 to 1.48 ± 0.23, p < 0.05). In *db/db* mice, the hypotriglyceridemic effect of atorvastatin was enhanced when combined with both GRT and glyburide (from 2.77 ± 0.50 to 1.73 ± 0.35, p = 0.0002). Glyburide reduced the severity and pattern of steatotic lipid droplet accumulation from a mediovesicular type across all lobular areas, whilst combining GRT with glyburide reduced the abundance and severity of lipid droplet accumulation in the centri- and mediolobular areas. The combination of GRT, glyburide and atorvastatin reduced the abundance and severity of lipid accumulation and the intensity score compared to the administered drugs alone. The addition of either GRT or glyburide in combination with atorvastatin had no effect on blood glucose or lipid profiles, but significantly reduced lipid droplet accumulation.

## Introduction

In Africa, 19 million people are living with diabetes, of which 4.6 million people are affected in South Africa alone (1). In type 2 diabetes elevated glucose levels in conjunction with increased triglyceride (TG) levels, exacerbate insulin resistance, dyslipidemia and cellular damage that complicates treatment regimens. Insulin resistance and dyslipidemia can cause impaired cardiac function, cardiovascular disease (2) and atherosclerosis (3). The cumulative increase of cholesterol, triglycerides (TG), and low-density lipoproteins (LDL-C) are indicators of hepatic steatosis, leading to a potential lipotoxic state (3, 4), liver cirrhosis, and hepatocellular carcinoma (5). Therefore, statin therapy in conjunction with sulfonylureas is recommended for diabetic patients by most clinical practice guidelines (6).

Sulfonylureas are among the oldest drug classes in use for the management of type 2 diabetes. Sulfonylureas enhance insulin secretion in response to elevated glucose levels by blocking ATP-sensitive potassium channels in pancreatic beta cells (7). Improving glycemic control through the stimulation of insulin secretion also suppresses hepatic gluconeogenesis resulting in better glycemic control (8). *In vitro* studies of isolated human islets suggest that prolonged use of sulfonylureas may be toxic to beta cells, by inducing beta cell apoptosis and loss of beta cell mass (9). In clinical trials such as the U.K. Prospective Diabetes Study (UKPDS) and Diabetes Outcome Progression Trial (ADOPT), the findings showed that sulfonylureas initially increase early-phase insulin secretion in response to oral glucose tolerance tests, thus leading to a more rapid rate of deterioration of beta cell function and overall glycemic control compared to treatment with metformin, thiazolidinediones or insulin therapy (10, 11).

Statins lower LDL-C by competitively inhibiting 3-hydroxy-3-methyl-glutaryl coenzyme-A (HMG-CoA) reductase in the liver (12). Through the inhibition of the mevalonate pathway, statins also present with cholesterol-independent pleiotropic effects such as inhibiting macrophage inflammatory activity, endothelial cell function and vascular smooth muscle cell proliferation, as well as the reduction in several cellular biosynthetic pathways including those involved in glucose homeostasis (13–15).

The effective long-term management of T2D remains a therapeutic challenge. Monotherapies supplemented with natural medicines or phytoconstituents have shown appreciable improvements in the blood glucose levels in diabetic patients, as they interact with multiple pathways simultaneously (16).

Polyphenols are known to have anti-diabetic and lipid-lowering activities (17, 18). In addition, these biologically active compounds not only protect vulnerable cells such as pancreatic beta cells against increased oxidative stress and inflammation associated with insulin resistance and obesity, but they also affect genes and proteins that regulate both glucose and lipid metabolic pathways (19, 20). The glucose-lowering effects of rooibos extracts and aspalathin (a *C-glucosyl dihydrochalcone*), its major compound, have been demonstrated *in vitro* (21–26) and *in vivo* (22, 23, 25–29). Using a crossover design study in humans, Francisco (30) found that when consuming a standardised fat meal with commercial soda (control group) or a rooibos beverage (treatment group), rooibos treatment lowered blood glucose levels at 2 hours and 6 hours (-22% and -18%, respectively) post-ingestion when compared to the baseline (T = 0) value. Marnewick et al. (31) studied the effects of rooibos on oxidative stress and biochemical parameters in adults at risk of cardiovascular disease over a 6-week study period. The study demonstrated that consuming six cups of rooibos tea per day for 6 weeks caused a 14.4% marked decrease in serum glucose levels although this was not statistically significant.

Given the growing interest in the health-promoting effects of herbal products, including rooibos, herbal medicines and supplements are often used together with pharmaceutical therapeutics and have been estimated to be as high as 35%. This study, therefore, aimed to determine the effects of combining a pharmaceutical-grade green rooibos extract (GRT) with the hypoglycemic drug, glyburide, and the dyslipidemic drug, atorvastatin in a diabetic *db/db* mouse model.

## Materials and methods

### Rooibos extract

A pharmaceutical-grade aspalathin-rich green rooibos extract (GRT), containing 12% aspalathin, was used in the study. Characterization of its Z-2-(β-D-glucopyranosyloxy)-3-phenylpropenoic acid content and that of the major flavonoids was described previously (32, 33).

### Animals and diet

Male 6-week-old *db/db* mice were bred and housed at the Primate Unit Delft Animal Centre (PUDAC) under a 24 hr light/dark cycle in a temperature-controlled room with food and water *ad libitum*. The Ethics Committee for Research on Animals (ECRA) of the South African Medical Research Council (SAMRC) approved all procedures involving animals (ECRA approval reference 04/15). Mice were divided into 8 experimental groups (n = 6 per group), receiving GRT (100 mg/kg BW), glyburide (5 mg/kg BW), and atorvastatin (80 mg/kg BW) as mono-, co-therapies of GRT and atorvastatin, and the combination of GRT, atorvastatin, and glyburide. *Db/db* and *db^+^* (BKS.Cg-*Dock7^m^* +/+*Lepr^db^* J) controls received 0.1% dimethyl sulfoxide and Dulbecco’s phosphate-buffered saline. Mice were treated daily for 5 weeks. Bodyweight and fasting blood glucose (FPG) measurements *via* tail prick using a glucometer (One-Touch Select, M-Kem pharmacy, South Africa) were determined weekly. After 3 weeks of treatment, an intraperitoneal glucose tolerance test was conducted by injecting mice peritoneally with 0.2 g of glucose/ml/100 g bodyweight. Liver tissues were excised, weighed, and collected. Tissues collected for histology were fixed in formalin, embedded, and stained for further analyses. For gene expression analyses, tissues were stored in RNAlater.

### mRNA expression

Total RNA was extracted from mouse liver tissue using the RNeasy kit (ThermoFischer Scientific Inc., Waltham, MA, USA). Tissues were homogenised using a TissueLyser (Qiagen GmbH, Hilden, Germany), centrifuged at 13,500 g for 3 min, and the extracted RNA purified using the RNeasy kit according to the manufacturer’s instructions. RNA concentration and purity were quantified using a Nanodrop One spectrophotometer (Thermo Electron Scientific Instruments LLC, Madison, WI, USA). The RNA quality was determined using an Agilent 2100 Bioanalyser (Agilent Technologies, Santa Clara, CA, USA) where the inclusion criterium was RIN > 7. A Turbo DNase kit (ThermoFischer Scientific Inc. Waltham, MA, USA) was used to remove genomic DNA. RNA samples were converted to cDNA using the High-Capacity Reverse Transcription Kit (Applied Biosystems, Foster City, CA, USA). Quantitative real-time PCR was performed on the ABI 7500 Instrument (ThermoFischer Scientific Inc. Waltham, MA, USA) using the standard curve method. Predesigned and optimised TaqMan gene expression probes for *Apoa1* (Mm00437569_m1), *Fasn* (Mm00662319_m1), *Pparγ* (Mm00440940_m1), *Pparα* (Mm00440939_m1), *Scd1* (Mm00772290_m1), *Srebp1* (Mm00550338_m1), *Gsk3β* (Mm00444911_m1), *Fabp1* (Mm00444340_m1), *Irs2* (Mm03038438_m1), *Slc2a2* (Mm00446229_m1) were used for differential gene expression (ThermoFisher Scientific Inc. Waltham, MA, USA). Gene expression data were normalised to β-actin and hypoxanthine-guanine phosphoribosyltransferase (*HPRT*) as housekeeping genes.

### Biochemistry measurements

Blood samples were collected in BD Vacutainer^®^ SST gel tubes, centrifuged at 4000 rpm for 15 mins at 4 °C and the sera stored at -80˚C until assayed. To determine total cholesterol, high-density lipoprotein cholesterol (HDL-C), LDL-C, and TG contents, all samples were analyzed by Pathcare (Dietrich, Voigt, Mia & Partners, N1 City, Cape Town, SA), a pathology laboratory accredited by the South African National Accreditation System (SANAS). Briefly, total cholesterol was determined using the cholesterol esterase enzymatic method. A coupled enzymatic reaction using adenosine triphosphate (ATP) as an agent was used to determine the triglyceride (TG) content. A cholesterol esterase/cholesterol oxidase method, based on an enzyme chromogen system for quantification, was used to determine high-density (HDL-C) and low-density lipoprotein cholesterol (LDL-C) content, respectively.

### Histological assessment

Formalin fixed paraffin embedded sections of liver tissue were stained with hematoxylin-eosin (H&E) and scored histologically for steatotic changes. Hepatic steatosis of the obese diabetic *db/db* mice was assessed histologically by an experienced histologist, blinded to the treatment groups, using a steatotic severity scoring system adapted from Trak-Smayra et al. (34) and Liang et al. (35). Steatosis was assessed for lipid accumulation in the liver lobules by type (microvesicular, presence of minute cytoplasmic lipid droplets around a centrally positioned nucleus; mediovesicular, several medium-sized lipid vacuoles present in the cytoplasm of hepatocytes; and macrovacuolar, single large cytoplasmic lipid vacuole displacing the nucleus to the periphery of the hepatocytes), grade or severity [0 *<* 5%, grade 1 (5–33%), grade 2 (34–66%) and grade 3 (*>* 66%), zonal predominance [periportal (zone 1), mediolobular (zone 2) or centrilobular (zone 3)]. Samples were blind-coded and randomly assessed to avoid observational bias by the histologist.

### Statistical analysis

Data are presented as the means ± SEM and were compared using one-way ANOVA with Tukey and Dunnett’s *post-hoc* tests with p < 0.05 considered statistically significant. Statistical analyses were performed using GraphPad Prism^®^ version 7.03 (GraphPad Software Inc., San Diego, CA, United States).

## Results

### Blood glucose levels

**Figure 1:**
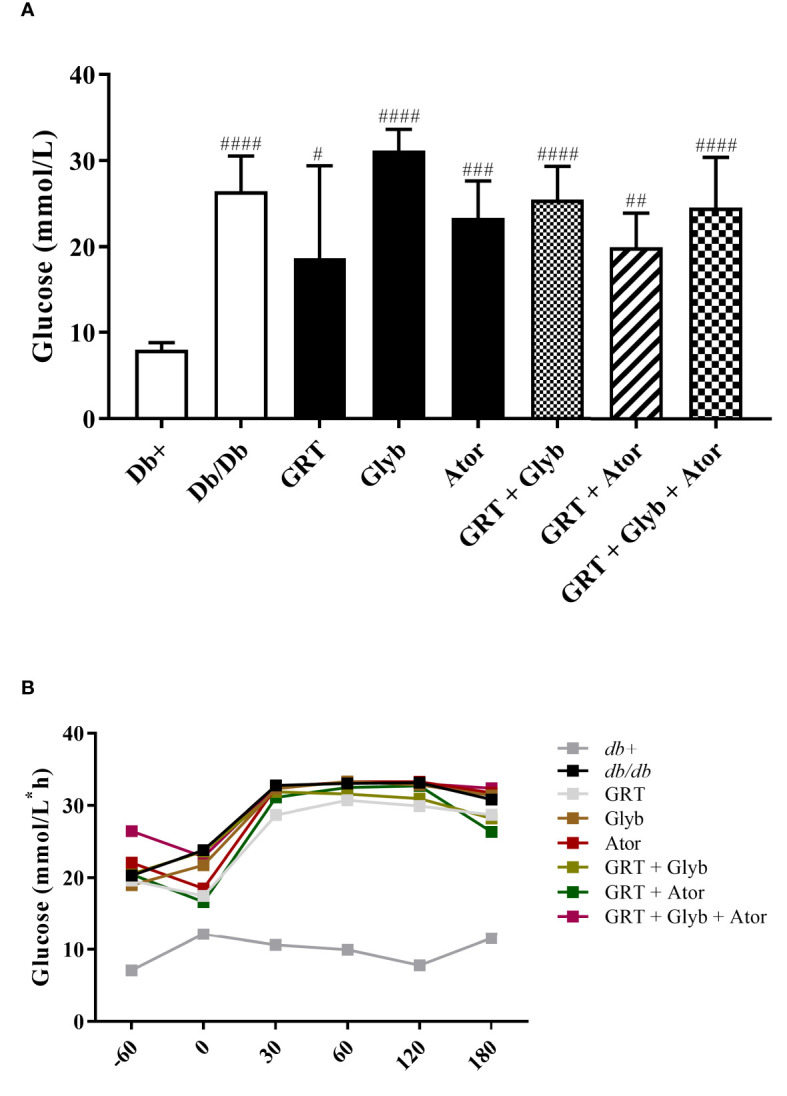
The effect of GRT, Glyb and Ator mono - or combination therapy on glycaemia as assessed by **(A)** fasting plasma glucose (FPG) and **(B)** intraperitoneal glucose tolerance test (IPGTT). IPGTT and FPG were assessed after 3 and 5 weeks of treatment, respectively. N = 5-6/group. ^#^p < 0.05; ^##^p < 0.01 and ^####^p < 0.0001 vs *db^+^* (One-Way ANOVA followed by Tukey post-hoc test). GRT, green rooibos extract; Glyb, glyburide; and Ator, atorvastatin.

The effects of GRT as either a mono- and/or co-treatment with glyburide, and/or atorvastatin, were orally tested in 6-week-old *db/db* mice. Fasting blood glucose levels of obese *db/db* (control) mice were significantly increased compared to their lean (*db* ^+^) counterparts (from 7.98 ± 0.83 to 26.44 ± 1.84, p < 0.0001) (**Figure 1A**). Interestingly, after 5 weeks of treatment, glyburide alone did not improve fasting blood glucose levels nor intraperitoneal glucose tolerance (p = 0.129), or in combination with GRT and/or atorvastatin (p = 0.818, **Figures 1A, B**), respectively.

### Body, liver, and adipose weight

The body, liver, and retroperitoneal fat (RF) weights of the obese *db/db* mice were significantly increased compared to their lean *db* ^+^ littermates (**Table 1**). GRT (from 1.35 ± 0.35 to 0.67 ± 0.04, p < 0.001) and glyburide (from 1.35 ± 0.35 to 0.78 ± 0.05, p < 0.001) monotherapy, and GRT with glyburide and atorvastatin (from 1.35 ± 0.35 to 0.48 ± 0.02, p < 0.0001) as a combined therapy significantly reduced RF weights, without affecting body or liver weight (**Table 1**).

**Table 1:** Body, liver and retroperitoneal fat weights of mice treated with GRT, glyburide, and atorvastatin for 5 weeks.

	*db^+^*	*db/db*	GRT (100 mg/kg)	Glyb (5 mg/kg)	GRT (100 mg/kg) + Glyb (5 mg/kg)	Ator (80 mg/kg)	GRT (100 mg/kg) + Ator (80 mg/kg)	GRT (100 mg/kg) + Glyb(25 mg/kg) + Ator (80 mg/kg)
**Bodyweight (g)**	25.00 ± 0.86	40.36 ± 1.33^****^	42.32 ± 0.82^####^	38.83 ± 0.98^####^	39.30 ± 1.19^####^	40.00 ± 0.67^####^	41.70 ± 0.82^####^	38.60 ± 1.75^####^
**Liver (g)**	1.35 ± 0.04	2.46 ± 0.07^****^	2.67 ± 0.13^####^	2.40 ± 0.08^####^	2.41 ± 0.16^####^	2.41 ± 0.08^####^	2.69 ± 0.04^####^	2.34 ± 0.09^####^
**RF (g)**	0.09 ± 0.01	1.35 ± 0.35^****^	0.67 ± 0.04^**,#^	0.78 ± 0.05^**,####^	0.47 ± 0.02^****^	0.45 ± 0.04^****^	0.51 ± 0.05^****^	0.48 ± 0.02^****^

Values are presented as the mean ± SEM of bodyweight (g), liver weight, and RF weight. Abbreviations: GRT, green rooibos extract; Glyb, glyburide; Ator, atorvastatin; RF, retroperitoneal fat. N = 5-6. ^#^p < 0.05; ^####^p < 0.0001 vs *db^+^*; ^**^p < 0.05; ^**^p < 0.001; ^****^p < 0.0001 vs *db/db* using One-Way ANOVA followed by Tukey *post-hoc* test.

### Blood lipid levels

**Figure 2:**
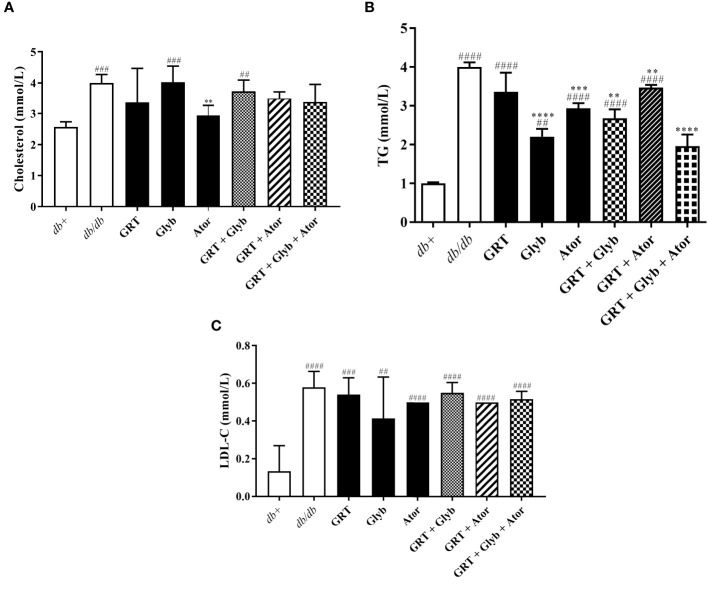
The effect of GRT, Glyb, and Ator mono- and combination treatments on serum lipid contents. Serum lipid contents of **(A)** cholesterol, **(B)** TG, **(C)** LDL-C were measured after 5 weeks of treatment. N = 5-6/group. ^##^p < 0.01, ^###^p < 0.0001 and ^####^p < 0.0001 denotes treatment vs db+ (non-diabetic control); **p < 0.01, ***p < 0.0001, and ****p < 0.0001 denotes treatment vs db/db (diabetic control)(One-Way ANOVA followed by Tukey post-hoc test). Abbreviations: TG, triglyceride; LDL-C, low density lipoprotein cholesterol, GRT, green rooibos extract; Glyb, glyburide; and Ator, atorvastatin.

Following treatment, serum TG, cholesterol, and LDL-C levels were significantly increased in *db/db* mice compared to the *db* ^+^ mice (**Figures 2A–C**). Atorvastatin expectedly reduced total cholesterol (from 4.00 ± 0.12 to 2.93 ± 0.13, p < 0.0001) and TG (from 2.77 ± 0.50 to 1.48 ± 0.23, p < 0.0001) compared to the untreated control (**Figures 2A, B**). In combination, GRT and atorvastatin treatment reduced TG levels (from 2.77 ± 0.50 to 2.05 ± 0.20, p < 0.01, **Figure 2B**). GRT monotherapy, the combination of GRT with glyburide, and GRT combined with glyburide and atorvastatin, reduced serum TGs (from 2.77 ± 0.50 to 1.73 ± 0.35, p = 0.001; 2.77 ± 0.50 to 2.68 ± 0.23, p = 0.0002; and 2.77 ± 0.50 to 1.96 ± 0.30) levels (**Figure 2B**).

### Hepatic glucose and lipid gene expression

mRNA expression of genes involved in lipid metabolism, lipid transport, insulin signalling, glucose metabolism, and lipogenesis in the liver were investigated. GRT, glyburide and atorvastatin monotherapies did not affect the expression of selected genes presented in **Table 2**. GRT in combination with atorvastatin significantly upregulated the expression of *ApoA1* (8.3-fold, p = 0.0006), *Fabp* (11.5-fold, p = 0.006), *Fasn* (5.4-fold, p < 0.05), *Gsk3β* (4.6-fold, p = 0.002), *Irs2* (4.3-fold, p = 0.003), *Pparγ* (27.9-fold, p = 0.009), *Pparα* (11.8-fold, p = 0.002), *Scd1* (10.0-fold, p = 0.004), *Srebp1* (11.5-fold, p = 0.004), and *Scl2a2* (6.8-fold, p = 0.0001) (**Table 2**). Whereas GRT combined with glyburide and atorvastatin increased the expression of *Fasn* (5.4-fold, p = 0.029) only (**Table 2**).

**Table 2:** Summary of gene expression of the liver of *db/db* mice.

Groups	*db^+^*	*db/db*	GRT (100 mg/kg)	Glyb (5 mg/kg)	GRT (100 mg/kg) + Glyb (5 mg/kg)	Ator (80 mg/kg)	GRT (100 mg/kg) + Ator (80 mg/kg)	GRT (100 mg/kg) + Glyb (5 mg/kg) + Ator (80 mg/kg)
***Apoa1***	0.2	4.9	↓ 2.2	↓ 4.7	1.3	↓ 2.2	↑ 8.3^***^	↓ 1.7
***Fabp***	0.6	1.6	1.2	↑ 3.3	↑ 1.9	1.4	↑ 11.5^***^	↑ 1.8
***Fasn***	0.6	1.8	↑ 2.9	↑ 2.4	1.3	↑ 3.1	↑ 5.4^*^	↑ 5.4
***Gsk3β***	0.9	1.0	1.4	1.4	↑ 3.1	↑ 5.5	↑ 4.6^**^	1.5
***Irs2***	0.5	2.1	1.3	↓ 1.6	↑ 5.8	↑ 4.6	↑ 4.3^**^	1.1
***Pparγ***	0.3	3.9	1.4	↓ 2.3	1.3	↓ 1.6	↑ 27.9^**^	↓ 3.7
***Pparα***	1.1	1.4	1.3	↑ 3.9	1.1	1.5	↑ 11.8^**^	↑ 2.2
***Scd1***	0.3	3.2	↑ 3.3	↑ 9.9	1.3	1.2	↑ 10.0^**^	↓ 2.8
***Srebp1***	0.7	1.4	1.3	↑ 4.8	↑ 1.8	1.4	↑ 11.5^**^	↑ 2.4
***Slc2a2***	0.7	1.5	1.3	↑ 1.9	1.4	1.5	↑ 6.8^***^	↑ 1.7

Gene fold-expression data normalised to β-actin and HPRT. N = 5-6. ^*^p < 0.05, ^**^p < 0.01, and ^***^p < 0.0001 vs *db/db* (One-Way ANOVA followed by Tukey *post-hoc* test). GRT, green rooibos extract; Glyb, glyburide; Ator, atorvastatin. ↓ arrow: down-regulation; ↑ arrow: upregulation.

### Histological scoring

**Figure 3:**
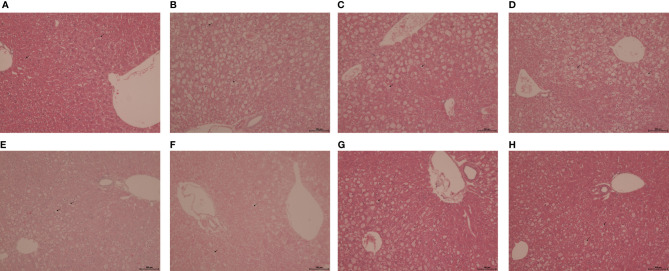
Representative H&E-stained liver sections from 11-week-old lean *db^+^* and obese *db/db* mice treated with **(A)** *db^+^* control, **(B)** *db/db* control, **(C)** GRT (100 mg/kg BW), **(D)** Glyb (5 mg/kg BW), **(E)** Ator (80 mg/kg BW), **(F)** GRT with Glyb, **(G)** GRT with Ator, and **(H)** GRT with Glyb, and Ator, respectively, for 5 weeks. N = 5-6/group. Microvesicular steatosis represented by a bold arrow and mediovesicular steatosis by a dotted line arrow. Magnification x 200; scale bar 100 μm. GRT, green rooibos extract; Glyb, glyburide; and Ator, atorvastatin.

The histological scoring of steatotic severity and type in untreated obese *db/db* mice confirmed the predominance of mediovesicular steatosis pattern (intensity score of 3) present in all hepatic acinar lobular zonal areas. After 5 weeks of treatment, GRT and atorvastatin reduced the scoring intensity from a predominantly mediovesicular lipid droplet type, to a mixed micro- and mediovesicular pattern (intensity score of 2), limited to the centrilobular (zone 3) and mediolobular (zone 2) areas. Glyburide treatment had no effect on the zonal severity and type of lipid steatosis, whilst combining GRT with glyburide reduced the abundance and severity to a mixed micro- and mediovesicular lipid droplet pattern mostly limited to the centrilobular (zone 3) and mediolobular (zone 2) areas. Similarly, the combination of GRT, glyburide and atorvastatin reduced the abundance and severity of lipid accumulation as well as the intensity score compared to monotherapies alone (**Table 3** and **Figure 3**).

**Table 3:** Histological steatotic scoring.

		Treatment groups						
		*db/db*	GRT (100 mg/kg)	Glyb (5 mg/kg)	Ator (80 mg/kg)	GRT (100 mg/kg) + Glyb (5 mg/kg)	GRT (100 mg/kg) + Ator (80 mg/kg)	GRT (100 mg/kg) + Glyb (5 mg/kg) + Ator (80 mg/kg)
**Microvesicular steatosis**	**Central (zone 3)**	-	1	1	1	1	1	1
	**Portal (zone 1)**	-	-	-	-	1	-	1
**Mediovesicular steatosis**	**Central (zone 3)**	3	2	3	2	2	2	2
	**Portal (zone 1)**	2	-	2	-	2	–	1
**Macrovacuolar steatosis**	**Central (zone 3)**	1	-	1	-	-	-	-
	**Portal (zone 1)**	-	-	-	-	-	-	-

Results for the histological steatosis score based on Trak-Smayra et al. (2011) (34) and Liang et al. (2014) (35) are presented. Score 0: < 5%; Score 1: 5-33%; Score 2: 34-66%; Score 3: > 66%. GRT, green rooibos extract; Glyb, glyburide; Ator, atorvastatin. N = 5-6.

## Discussion

Diabetic dyslipidemia presents with increased TGs, low HDL-C, and high LDL-C levels, often necessitating the combination of oral hypoglycemic drugs with lipid-lowering drugs to improve the clinical outcomes of type 2 diabetic patients. Hypertriglyceridemia is common in patients with T2D and places them at increased risk of CVD. In addition, hypertriglyceridemia is strongly associated with a host of other potential risk factors, including obesity, insulin resistance and increased levels of apolipoprotein (36). In rodents, increased rates of fatty acid synthesis lead to the development of hepatic steatosis. In an insulin-resistant state, the influx of fatty acids from adipocytes increases *de novo* lipogenesis whilst decreasing fatty acid oxidation, resulting in TG accumulation in the liver (37). Combination therapies have proven more effective in treating multi-factorial metabolic conditions, including the management of lipid disorders.

Glyburide, belonging to the sulfonylurea class of insulin secretagogues, lowers blood glucose levels by stimulating beta cells to produce and secrete more insulin (38). Our results showed that glyburide alone or combined with GRT and/or atorvastatin did not affect blood glucose levels of obese *db/db* mice. This finding is inconsistent with other studies in diabetic animals and humans that showed reductions of FPG levels in glyburide-treated groups (39–42). In diabetic Wistar rats, Neerati and Gade (43) observed that combined administration of glyburide with atorvastatin enhanced the reduction in blood glucose levels. Albeit in different models, one such study showed that after 4 weeks of treatment, glyburide at 5 mg/kg reduced FPG in an alloxan-induced diabetic mouse model as well as improved the clearance of blood glucose at each time point monitored versus vehicle-treated diabetic mice (42). *Db/db* mice are obese, highly insulin - and leptin-resistant and spontaneously and progressively develop worsening diabetes over time culminating in beta cell mass depletion. Hence, the persistent increased demand for insulin leads to the hyperactivity of beta cells which in the long-term, results in a dysfunctional secretory response to glucose (44), thus providing a possible explanation for the lack of effect of glyburide observed in this study. Surprisingly, however, the combined effect of GRT and atorvastatin improved glucose levels in these obese *db/db* mice. This finding could be attributed to the effect of GRT, in improving inflammatory conditions and insulin resistance. There is accumulating evidence to suggest an emerging role of inflammation as an early driving factor for the development of insulin resistance and type 2 diabetes. Moreover, treatment of obese mice with statins over several weeks was sufficient to decrease insulin-induced glucose uptake in adipose tissue. Importantly, treatment with statins lead to dysregulated insulin signaling in explanted adipose tissue, whilst insulin signaling was maintained in the adipose tissue of mice deficient in NLR Family Pyrin Domain Containing 3 (NLRP3), and in explants treated with glyburide (45), a NLRP3 inflammasome inhibitor.

Atorvastatin is currently the most-prescribed cholesterol-lowering agent for treating elevated levels of LDL-C, TG, and cholesterol. Although statins lower cardiovascular risk, they may also promote adverse effects such as new-onset diabetes, myopathy, rhabdomyolysis, and induce hepatotoxicity (46, 47). As expected, in this obese diabetic *db/db* mouse model atorvastatin monotherapy reduced TG levels. This is in accordance with literature that showed reduced TG levels of atorvastatin administered at either low (10 mg/kg) or high doses (80 mg/kg) (48, 49). The enhanced decrease in TG levels induced by glyburide co-therapy may, at least in part, be due to the glyburide-induced insulin release that activated lipoprotein-lipase and the hydrolysis of TGs (40). This effect was further enhanced in combination with GRT and glyburide. Co-administration of GRT with atorvastatin did not improve cholesterol, LDL-cholesterol, or TG levels relative to atorvastatin alone. With the addition of glyburide to GRT and atorvastatin, an additive reduction in TG levels was observed. In part, the enhanced effect could be due to the drug-drug or herb-drug interactions of glyburide and/or GRT on the pharmacokinetics of atorvastatin, respectively. This was evidenced by decreasing the metabolic clearance and increasing the C_max_ of glyburide thereby effectively increasing its therapeutic effect (43). Previously, we showed that GRT inhibits the activity of CYP2C9 and CYP3A4, enzymes involved in the metabolism of glyburide and atorvastatin, suggesting that further herb-drug interactions are likely when GRT is co-administered with glyburide and atorvastatin (33). This could have added to the improvement of glycemia by the combination of GRT and atorvastatin. In addition, the added positive effects on body and liver weight together with lower TG levels induced by the combination of GRT, glyburide and atorvastatin are an important outcome of this study.

In the liver, statin treatment is associated with worsening glycemic control (50) and a small incremental increase in fasting blood glucose levels (51). A known characteristic of insulin resistance and T2D is the elevation of hepatic gluconeogenesis contributing to hyperglycemia (52). Atorvastatin treatment upregulates thyroid hormone-responsive spot 14 protein (THRSP) expression, a small protein found in the liver that is predominantly expressed in lipid-producing tissues. This protein has been implicated in the regulation of lipogenic processes by controlling the expression of fatty acid synthase (*Fasn)* and sterol regulatory element binding protein (*Srebp)* lipogenic genes (53, 54), thereby increasing free fatty acids (FFAs) in the liver, which can contribute to the development of steatosis. In this study, atorvastatin reduced mediovesicular lipid accumulation around the centrilobular area, whilst significantly increasing both *Fasn* and *Srebp* gene expression when combined with GRT. *Srebp* regulates hepatic *de novo* lipogenesis by insulin and mediates the synthesis of fatty acids and TGs (37). In rodent models of insulin resistance and obesity, increased rates of hepatic fatty acid synthesis contribute to the development of hepatic steatosis (55). There is a direct relationship between body fat levels, leptin, and insulin resistance, with the leptin receptor deficient *db/db* mouse presenting with alterations in lipid metabolism and liver function including steatosis (56). Hence hyperleptinemia could account for the resultant increase in *Srebp* expression lipogenesis and steatosis. In steatotic livers, stearoyl-CoA desaturase (*Scd*) regulates monounsaturated fatty acid (MUFA) synthesis whilst preventing the progression of steatosis to non-alcoholic steatohepatitis (NASH). High *Scd1* expression is correlated with metabolic diseases such as obesity and insulin resistance, whereas low levels are protective against these metabolic disturbances (57). *Scd1* protection is through channeling saturated fatty acids into MUFAs, which are easily incorporated into TGs (58). The reduction in FPG, TGs and lipid accumulation observed within the same treatment group could lessen steatosis and decrease insulin resistance. A study by Layman et al. (59) also showed that GRT alone displayed hepatoprotective effects by reducing hepatic steatosis.

Furthermore, *Slc2a2* and *Irs2* were both upregulated by this combined treatment group, coupled with the reduction in FPG, which could infer an increase in insulin signaling by the activation of IRS/PI3K/AKT in the liver. A study reported that the abnormal accumulation of TG in the diabetic liver is due to the simultaneous activation of lipogenesis and gluconeogenesis, leading to excessive lipid production (60). *PPARα*, a transcriptional factor predominantly expressed in the liver, plays a key role in maintaining lipid homeostasis through the regulation of various enzymes in lipid and glucose metabolism (61). The current study demonstrated that GRT combined with atorvastatin upregulated the mRNA expression of *PPARα* in the liver of obese diabetic *db/db* mice. The modulating effects of *PPARα* on lipid metabolism and inflammation may explain our finding that *PPARα* activation modulated dyslipidemia in this diabetic mouse model.

Despite the unexplained increase in some hepatic lipogenic genes in these obese diabetic *db/db* mice, the reduction in TGs by GRT and atorvastatin is favorable. Clinically, although statins can harm glycemia, the suppression of hypertriglyceridemia and the reduction in steatosis could help to improve glycemic control in patients with T2D (62). This study suggests that the addition of GRT enhances therapeutic potential in the obese diabetic *db/db* mouse, however, clinical studies are needed to further validate these findings. Moreover, in the current study, glyburide failed to influence glucose levels, and as glyburide is an NLRP3 inflammasome inhibitor, the combined effect of glyburide and statins on adipose tissue should therefore be further explored.

## Conclusion

This study demonstrated that co-therapy of GRT with atorvastatin and/or glyburide, enhanced the glucose and lipid-lowering effects of obese diabetic *db/db* mice, which was associated with effects on hepatic steatosis and retroperitoneal fat accumulation. These results further demonstrate that the combination of GRT together with conventional treatments such as hypoglycemic and hypolipidemic medications may have beneficial effects on type 2 diabetes.

## Data availability statement

The original contributions presented in the study are included in the article/supplementary files, further inquiries can be directed to the corresponding author.

## Ethics statement

This animal study was reviewed and approved by the Ethics Committee for Research on Animals (ECRA) of the South African Medical Research Council. The study was carried out in accordance with the recommendations of the Ethics Committee for Research on Animals (ECRA) of the SouthAfrican Medical Research Council (ref. 04/15).

## Author contributions

OP, CM, EJ, BR, and CA participated in research design. OP conducted laboratory experiments. OP and CM performed data analysis. OP, CM, EJ, BR, and CA wrote or contributed to the writing of the manuscript. All authors contributed to the article and approved the submitted version.

## Funding

This research was funded in part by the National Research Foundation (NRF) Thuthuka Programme (Grant 99381 to OP), the Japan Society for the Promotion of Science/NRF Research Cooperation Programme (NRF Grant 108667 to EJ) and the Biomedical Research and Innovation Platform of the South African Medical Research Council. Afriplex GRT™ was provided by Afriplex, Paarl, South Africa.

## Acknowledgments

Special thanks to the staff of PUDAC and BRIP, especially Joritha van Heerden and Desmond Linden for their assistance with the animal work.

## Conflict of interest

The authors declare that the research was conducted in the absence of any commercial or financial relationships that could be construed as a potential conflict of interest.

## Publisher’s note

All claims expressed in this article are solely those of the authors and do not necessarily represent those of their affiliated organizations, or those of the publisher, the editors and the reviewers. Any product that may be evaluated in this article, or claim that may be made by its manufacturer, is not guaranteed or endorsed by the publisher.
